# ‘What are nurses’ and healthcare workers’ cultural understandings of dementia?’ An integrative literature review

**DOI:** 10.1177/14713012241285525

**Published:** 2024-09-21

**Authors:** Catharine Jenkins, Analisa Smythe

**Affiliations:** 1725 Birmingham City University, UK; Birmingham and Solihull Foundation Mental Health Trust, UK; The Royal Wolverhampton NHS Trust, UK

**Keywords:** transcultural, dementia, nurses and healthcare workers, explanatory models, global

## Abstract

**Aim:** to explore the range of cultural understandings of dementia held by people providing nursing care globally.**Background:** There is a worldwide shortage of nurses and healthcare workers, resulting in extensive global mobility among the workforce. Cultural competence is expected of nurses who serve diverse populations and although self-awareness is recognised as crucial in developing this ability, the focus has tended to be on the identity of the patient and adjusting care according to their specific needs. However, taken-for-granted assumptions drive unconscious judgements and therefore behaviour so nurses’ dementia-related understandings are worthy of exploration.**Design:** An integrative literature review, comprising five stages: problem identification; literature search; data evaluation; data analysis; and presentation of the findings.**Methods:** Six databases were searched for original research published between 1997 and 2023. Studies which focus on qualified/registered and unqualified/unregistered healthcare workers’ cultural understandings of dementia were included. Studies were evaluated using a tool designed for the critical assessment of qualitative research. Data was extracted using a bespoke spreadsheet. Conventional content analysis was undertaken to develop a synthesised summary of the findings of the studies.**Findings:** 11 papers met the inclusion criteria. Content analysis led to identification of two main themes: ‘Stigma as a common factor in cultural perceptions of dementia’, and ‘Stigma derived from cultural perceptions has consequences for people living with dementia’.**Conclusion:** An international perspective facilitated insight into alternative perceptions of the nature of dementia and care responses. A version of the ‘Relationship Centred Care’ model, expanded to include the wider community, could support theoretical and practical recommendations for culturally congruent approaches to care. Further research is required to examine the usefulness of incorporating this approach internationally.**Reporting Method:** The authors followed the ENTREQ reporting guidelines (Tong et al., 2012).

## Introduction

The number of people living with dementia continues to rise, with the most significant increases in low- and middle-income countries (World Health Organisation (WHO) 2017). Dementia affects the practical, emotional, communicational, and interpersonal functions of those that live with it, and has an impact not only on individuals, but also on their families, communities and governments worldwide (World Health Organisation, 2012). Lay understandings of dementia in high income countries tend to focus on poor short-term memory and associate this with normal ageing (Nagel et al., 2021), while the medical explanatory model leads to a focus on specific disease processes (Burton & Power, 2018), and nurses in high income countries are encouraged take a person-centred bio-psycho-social approach (Gibson, 2017). More recently dementia been framed as a disability, for which adjustments should be made (Cahill, 2022). Nurses may belong to different (personal and professional) cultures simultaneously and so share both lay and professional beliefs. Many nurses are bi-cultural, experiencing a process of acculturation to the values of a second homeland and all are acculturated to the values and beliefs of their profession through nurse education (Mariet, 2016) and the predominant workforce culture of their employment (Feltrin et al., 2018).

Cultural beliefs and values can be framed (Hofstede, 2001) according to ‘dimensions’, such as ‘individualism-collectivism’ (the extent to which a person views the world as an individual or with emphasis on the group) and ‘power-distance’ (respect for hierarchical status in relationships) which vary on continuums at population levels. Increasing migrant populations worldwide mean this theory is relevant to nursing. There is a worldwide shortage of nurses and healthcare workers, which has contributed to extensive global mobility among the workforce (Drennan & Ross, 2019). Cultural competence is expected of nurses who serve diverse populations (Fadaeinia et al., 2022). Cultural competence is understood as the nurse being able to adapt care so that it is congruent with the cultural identity of the patient and family, and although self-awareness is recognised as crucial in developing this ability (Narayan & Mallinson, 2022) the focus has tended to be on the identity of the patient and adjusting care according to their specific needs (Anton-Solanos et al., 2022). However, taken-for-granted assumptions drive unconscious judgements and therefore behaviour (McMahon, 2020), so the range of nurses’ dementia-related understandings is worthy of exploration. Nurses’ and healthcare workers’ experiences are important too; moral distress can arise when care cannot be provided in ways the nurse feels are ethically right Spenceley et al. (2017). This review explores the range of cultural understandings of dementia held by nurses and healthcare workers worldwide, to identify alternative perspectives and explore contradictions, which may enable new understandings that challenge, build on and potentially replace the mix of folk beliefs and currently accepted theoretical orthodoxies that underpin education and practice.

The theoretical understandings that support a person-centred approach to care in the United Kingdom are drawn from the work of Tom Kitwood (1997); his ideas reflect his own cultural norms, as a white, male, straight, cis-gender, educated person living in a rich nation. They emphasise valuing the individual, while also challenging stigma and ageism. In the global north the underpinning philosophy and applications of his ideas in practice continue to be widely accepted as key to high-quality care (Terkelsen et al., 2020). People living with dementia themselves identify their priorities as being socially included, able to enjoy meaningful activities and relationships and maintaining physical health (Reilly et al., 2020), considerations which are congruent with Kitwood’s ideas but which may also echo broader culturally driven expectations.

Kitwood’s work involves some challenging concepts, for example that ‘Personhood’ (individual identity) is ‘bestowed’ on a person through their experiences of relationships with other people (Kitwood, 1997). His work is rarely critiqued even though practice realities may not match the rhetoric (Garratt et al., 2021) and his theories may not always chime with nurses’ own beliefs. For example, nurses may believe that ‘Personhood’ is intrinsic to being human, regardless of others’ behaviour, or alternatively believe that Personhood diminishes alongside the capacities associated with being a citizen such as the ability to be accountable for ones’ actions. The nature of nurses’ beliefs about caring for people with dementia may relate to quality of care provided and individuals’ attitudes are often identified as key (Kunz et al., 2022). Cultural self-awareness is part of cultural competence, so exploring values and norms associated with the person-centred model is relevant to the purpose of this review. However, it is important to also acknowledge the impact of structural factors such as funding, training opportunities and staffing levels which affect nurses’ provision of the excellent standards of care to which they aspire (Urashima et al., 2021).

The current authors have been educated in the person-centred model, followed its teachings in practice and used it to underpin their own research and educational activities. However, despite the congruence of the model with our own personal cultural and professional backgrounds, it is important to acknowledge the diversity of the nursing workforce, with its related range of dementia-related beliefs and norms, and recognise that acculturation can be a reciprocal, mutually beneficial learning experience. Alternative culturally situated perceptions could challenge the person-centred approach or provide insights which would enable its adjustment, to facilitate greater congruence for service-users and the workforce or provide the beginnings of a fresh understanding with an alternative or complementary care focus.

## The review

### Aim

The aim of this integrative literature review is to explore the range of cultural understandings of dementia held by people providing nursing care globally.

### Design

Whittemore and Knalf’s (2005), modified framework for research reviews guided an integrative review of the literature. There are five stages to this methodology: problem identification; literature search; evaluation of data; data analysis and presentation of findings. The review process was guided by a protocol. The review was registered with PROSPERO (CRD42023453065). The authors followed the ENTREQ reporting guidelines (Tong et al., 2012).

### Search methods

The following databases were searched:

• Allied and Contemporary Medicine Database (AMED)
• BNI
• CINAHL
• Emcare
• MEDLINE
• PsycINFO

The question “What are nurses’ and healthcare workers’ cultural understandings of dementia?” was used. The population was registered/qualified and unregistered/unqualified nursing healthcare workers. It was important to include unregistered nurses because in low-and middle-income countries nursing care is provided by workers who may not be formally educated and registered. Subsequently the concept was cultural understandings, and improving the understanding of dementia was the health care problem.

A range of search terms were identified with the support of a specialist librarian, to ensure relevant literature was retrieved. The search terms were combined with synonyms and Boolean operators OR and AND. The technique of truncation was also used.

### Inclusion and exclusion criteria

Studies were selected for inclusion based on inclusion criteria, guided by the review aim.

Inclusion Criteria:

i) Studies which focus on trained (qualified/registered) and untrained (unqualified/unregistered) healthcare workers’ (including lay workers) cultural understandings of dementia.
ii) Studies published between 1997- 2023, guided by the date of early literature around approaches to dementia care.
iii) Qualitative studies and mixed methods.

Exclusion Criteria:

i) Abstracts, discussion papers, commentaries, letters, editorials and grey literature such as dissertations and theses were excluded.
ii) Papers published before 1997, quantitative studies, studies exploring other healthcare professionals’ understandings (for example doctors and allied health professionals) and of other health conditions, plus papers not in English, or with no available translation, were also excluded.

### Search outcomes

**Figure 1.**
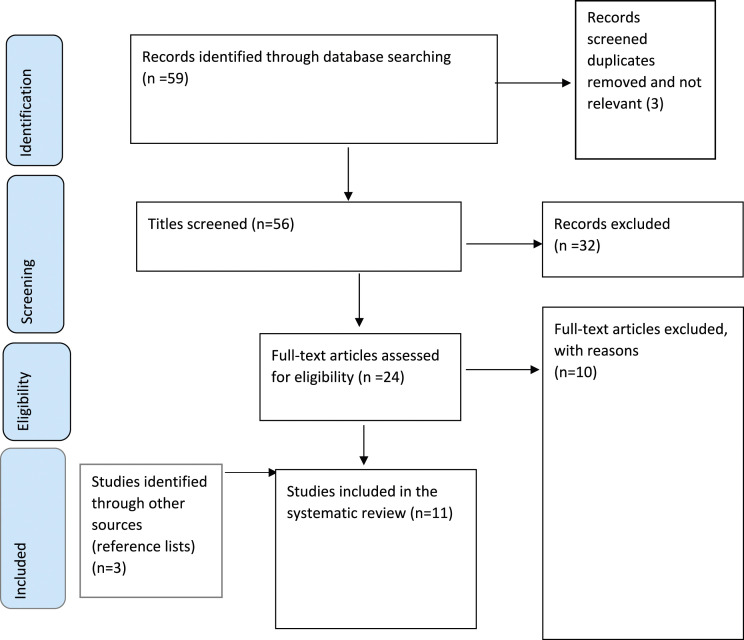
PRISMA flow diagram.

The total search identified 54 records, with non-relevant and duplicated papers removed. Titles were assessed for suitability. Subsequently abstracts were screened, followed by full text screening for relevance against the inclusion criteria of articles (*n* = 24). Both authors independently carried out abstract and title screening and read full texts. Following hand-searching of reference lists of the included studies, three additional papers met the inclusion criteria. A total of 11 papers were therefore included in the review. (Figure 1. PRISMA flow diagram).

### Quality appraisal

The authors evaluated the studies independently using the Hawker checklist (Hawker et al., 2002), a tool designed for the critical assessment of qualitative research. Each paper was reviewed by both authors. Most papers were high quality (30–36 points). Two papers were medium quality (24–29 points) (Karungi et al., 2022; Lee et al., 2018). No papers were low quality. The Hawker checklist (2002) was used to support the evaluation of the methodological quality of studies and their interpretation rather than their acceptance or rejection (Walker & Gavin, 2019). In the event of disagreement, issues were discussed and consensus achieved.

## Data extraction

A spreadsheet was used to record standard information: author, year, setting, study aim, sample, key findings and strengths and limitations. Please see data extraction table for study characteristics (Table 1. Data extraction table).

**Table 1.** Data extraction table.

Author, Year, Country	Study A	Sample/Setting	Design/Method	Main findings	Strengths/limitations
Antelius and Kiwi (2015) Sweden.	To explore how dementia can be understood and experienced by Iranian immigrants who may wish to work with people with dementia.	Participant observation and in-depth interviews. (*n* = 66). Field and data gathered in two separate settings a residential home and home help.	Content and ethnographic analysis.	There were four main findings related to ethnocultural dementia care. These include (a) a wider recognition of people from different CALD backgrounds possibly having different perceptions of what dementia is, (b) a possibility that such ascribed meaning of dementia has a bearing on health maintenance and health-seeking behaviour as well as the inclination to use formal services or not, (c) choosing to use formal service in the forms of ethnoculturally profiled dementia care facility seems to relate to being able to “live up to ideals of Iranian culture,” and (d) “culture,” however ambiguous and hotly debated a concept it is, appears to be a relevant aspect of people’s lives, an aspect that is both acquired as well as ascribed to oneself and to others.	The paper is rather difficult to navigate. The abstract lacks structure, there is no conclusion and strengths and limitations are not acknowledged. Strengths of the study included the large sample size which allows for a rich understanding of carers views of dementia within a cultural context. The method is a combination of content and ethnographic analysis which is appropriate and described clearly (e.g. questionnaires included).
Bentwich et al. (2018) Israel.	To explore carers attitudes towards the autonomy and human dignity of people with dementia.	A total of 20 staff from three different cultural groups in Israel (‘‘Sabras,’’ ‘‘Arabs,’’ and ‘‘Russians’’), working in nursing homes and a hospital.	A qualitative research approach was used, utilizing semi-structured interviews and thematic-phenomenological content analysis.	In comparison with the other groups, most Arab carers offer markedly richer perceptions of human dignity and autonomy. Their human dignity’s conceptualization emphasizes ‘‘person-centred approach,’’ and their perception of patients’ autonomy includes provision of explanations, preservation and encouragement of independence.	Limitations are not acknowledged. The generalisability of the findings is limited, the study was conducted in the northern part of Israel with 3 different cultural groups in Israel ‘Sabras,’’ ‘‘Arabs,’’ and ‘‘Russians’’ other cultural groups in Israel may have been omitted. The sample selection is also not clear. Strengths include a robust design with thematic phenomenological content analysis. Data was collected until a saturation was reached.
Egede-Nissen et al. (2017). Norway.	To explore the challenges of health care workers who work with people with dementia.	Five health care workers from minority backgrounds working in nursing homes.	Qualitative interviews using a narrative approach. Phenomenological hermeneutic method.	Findings: One theme (with four subthemes): striving to understand the quality of care for persons with dementia.	Limitations are not acknowledged. Strengths include a comprehensive review of the literature. The method is appropriate and clearly explained. The study contributes something different in terms of understanding with clear implications for patient care.
Hirata and Harvath (2016). Japan	To explore Japanese care workers’ attributions, beliefs and cultural explanations of physically and psychologically aggressive behaviour symptoms.	137 care workers in 10 nursing homes In the northern and western areas of Japan.	This study reports on the results of three open-ended questions that were part of a larger study that explored Japanese care workers’ experiences with aggressive behaviour symptoms in persons with dementia.	All causes for residents’ aggression that participants linked to care workers, were related to their approach. In the concept of joge (hierarchy), a younger person (me-shita) is supposed to respect an older person (me-ue).	The study uses qualitative data from an open-ended questionnaire which may have limited the depth of participants responses. The number of respondents is not clear and the ‘qualitative content analysis’ is brief and lacks detail. Limitations are acknowledged.
Lee et al. (2018) USA.	To explore experience of Korean American personal carers caring for older Korean Americans with dementia symptoms.	Four focus groups with 23 Korean American family caregivers and two focus groups with Ten Korean American carers of older Korean Americans with dementia symptoms.	Exploratory qualitative approach using a focus group approach.	Highlights the significance of culturally competent service delivery in dementia caregiving. Need for more education and training.	The abstract is unstructured, there is no information about the number of participants or how many focus groups were conducted. There is no ethics section and bias is not explored. The study used a small purposive sample therefore the findings of this study may not be generalisable to Korean Americans as a group. The study provides useful insights into assumptions about the causes of dementia.
Mazaheri et al. (2017) Sweden.	To “illuminate” the meaning of conscience by enrolled nurses with an Iranian background working in residential care for Persian-speaking people with dementia.	A total of ten enrolled nurses with Iranian background, in residential care settings for Persian speaking people with dementia in a large city, in Sweden.	A phenomenological hermeneutical method guided the study. Narrative interviews were used to collect the data.	Nurses strived to keep their conscience clear by being generous in helping others, accomplishing daily tasks well and behaving nicely in the hope of being treated the same way one day. Cultural frameworks and the context of practice needed to be considered in interpreting the meaning of conscience and clear conscience	More in-depth interviews could have enriched the findings, all participants were female and lived and worked in an urban area. Strengths include a clear and detailed methods section, although more examples of ‘probing questions’ would have been useful. The study uses a robust design, strengths and weaknesses are acknowledged.
Mkhonto et al. (2017) Tshwane in South Africa.	To explore and describe the link between culture and dementia care with the focus on the influence of the belief in dementia as witchcraft and people with dementia as witches.	One close family member and seven nurses caring for patients with severe dementia in nursing homes.	Findings from a larger international study is presented with in-depth qualitative interviews. A hermeneutic analytic approach was used.	The “witch” belief prevents seeking professional help. Awareness raising in churches, schools and clinics and facilitate support groups for carers of people with dementia in the local language.	The study aims are not clear. At times the paper is lacks clarity as some of the research relates to another larger study. The study included 37 participants, only participants who talked about witchcraft were included in the study (*n* = 8). The fundings are also limited with only two themes. in connection with dementia, these interviews offered rich data on this topic.
Musyimi et al. (2021) Kenya.	To explore perceptions towards dementia and related care across three stakeholder groups in rural Kenya.	A total of 38 key stakeholders (carers of persons with dementia from mental health, health care providers and the general public) Additionally five individual interviews were held with carers.	Qualitative. Focus groups	Across the three participant groups, a total of four themes were identified: (i) negative stereotypes of dementia, (ii) limited knowledge about dementia, (iii) diagnostic pathway and (iv) neglect and abuse.	The study may lack generalisability as it included a small group of individuals from rural Kenya. Data was included from both focus groups and individual interviews.
Nwakasi et al. (2021) Nigeria and USA.	To understand the everyday understanding of dementia and the impact of stigma on the caregiving experiences of informal female Nigerian dementia caregivers.	12 informal female caregivers in Nigeria. Findings were shared with 21 adult Nigerians residing in the USA for further insight on the findings.	This qualitative descriptive study used semi-structured interviews. Results were presented to focus groups of Nigerians residing in the United States.	The three major themes were misconceptions about dementia symptoms, caregiving protects against stigmatisation, and stigma affects caregiving support. Overall, we argue that knowledge deficit, poor awareness, and traditional spiritual beliefs combine to drive dementia-related stigmatisation in Nigeria.	The study includes data from interviews and focus groups, it is not clear how the data is combined for the purpose of analysis. The data does not appear to be rich enough to allow for new understandings of the phenomenon. Limitations are acknowledged. The interviews were conducted over the phone which may have limited the depth of responses.
Brooke et al. (2019) Higher Education Institutes in England, Slovenia and New Zealand.	To explore the cultural beliefs of dementia of student nurses.	Student nurses studying nursing in England (*n* = 81), Slovenia (*n* = 41), Philippines (*n* = 53) and New Zealand (*n* = 6). Participants from England and New Zealand were from diverse cultural backgrounds. Student nurses at the beginning of their studies (*n* = 100) and towards the end of their studies (*n* = 81) participated.	Focus Groups.	Two major themes were identified in the data: familial piety and dementia discourse. Familial piety emerged from the importance of family. Dementia discourse emerged from the terminology student nurses applied, such as: ‘preconceptions and misconceptions’ of aggression, and ‘considered crazy’ stigma of dementia due to a lack of awareness.	Limitations are acknowledged. The study includes specific countries (England, Slovenia, Philippines and New Zealand) which may limit the generalisability of the study. Another limitation was the invitation process, as all students in identified cohorts were invited, which may have resulted in participants with an interest in dementia volunteering, providing some bias in their experience and knowledge. Strengths include a robust design, multiple coders analysed the data, the study included students from three universities, reflexivity is also discussed, the authors of this paper were experienced healthcare professionals. The study adhered to guidelines for reporting qualitative research (CORECQ).
Karungi et al. (2022) Uganda.	To explore Lay Health Workers’ perceptions of a training intervention in rural southwestern Uganda in community-based care and management of people with dementia, and implementation of the knowledge and skills gained.	Community-Based Care 30 lay health workers were interviewed.	Prior to the intervention, a training needs assessment was conducted. Semi-structured interviews were conducted “post” intervention to assess the participants’ perceived knowledge and skills gained as well as the experience with the implementation process intervention.	Participants felt more at ease working with people with dementia and reported a notable change in the attitude of family members. They also reported challenges in differentiating the signs of early dementia from superstitious beliefs.	Limitations included the methods section which lacks clarity. Limitations are acknowledged. The study was a single district pilot intervention study therefore may not be generalizable. Interviews were conducted by the staff who delivered the training intervention which may have influenced the participants’ responses.

### Synthesis

Conventional content analysis (Hsieh & Shannon, 2005) was conducted independently by both authors, to synthesise findings of the studies. Content analysis consists of 4 stages a) becoming familiar with the data, an iterative process which involves questioning one’s own preconceptions and ideas b) highlighting the exact words from the text that appear to relate to key thoughts or concepts and subsequently summarising these meaning units c) formulating codes, d) developing categories and themes based on how the different codes are related and linked (Erlingsson & Brysiewicz, 2017; Hsieh & Shannon, 2005). This qualitative analytical approach is generally used with a design whose aim is to describe a phenomenon (Hsieh & Shannon, 2005), in this case cultural understandings of dementia.

In the findings section below, the literature is synthesised under two main themes.

## Findings

### Study characteristics

The papers all used qualitative methods; one-to-one interviews, observations, and focus groups (See data extraction table 1). Two studies were conducted in Sweden Antelius & Kiwi (2015); Mazaheri et al. (2017), one in Israel (Bentwich et al., 2018), one in Norway (Egede-Nissen et al., 2017), one in Japan (Hirata & Harvath, 2016), one in the United States of America (USA) (Lee et al., 2018), one in Uganda (Karungi et al., 2022) one in South Africa (Mkhonto & Hanssen, 2017) one in Kenya, (Musyimi et al., 2021), (which also included members of the public in separate focus groups, as this content did not match our eligibility criteria this element is not reported on in this paper) one in both the USA and Nigeria (Nwakasi et al., 2021) and another in the UK, Slovenia and New Zealand (Brooke et al., 2019).

Two themes emerged through data analysis: ‘Stigma as a common factor in cultural perceptions of dementia’, and ‘Stigma derived from cultural perceptions has consequences for people living with dementia’.

### Theme 1: Stigma as a common factor in cultural perceptions of dementia

**Diagram 1.**
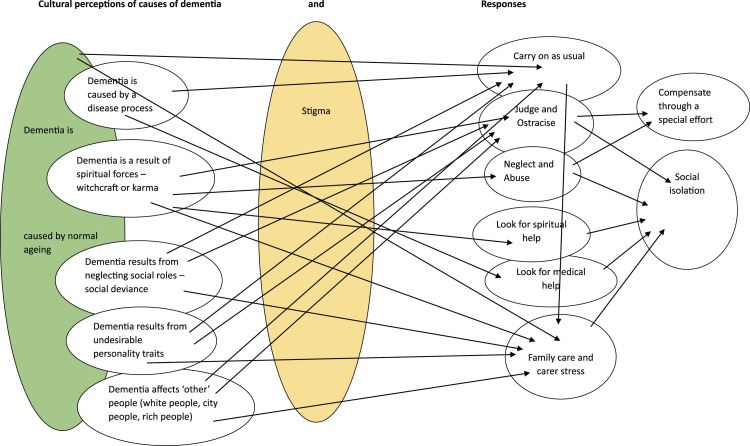
Overlapping themes.

This theme reflects spiritual, medical, ageing, social deviance and personality traits as causative factors in development of dementia. (See Diagram 1).

Seven of the eleven papers suggested that culture can shape understandings of the causes of dementia. Cultural perceptions of dementia in South, Central, West and East Africa were linked with spiritual beliefs such as witchcraft, with those diagnosed with dementia thought to be witches or bewitched by evil spells, this was particularly in relation to older or middle-aged women (Musyimi et al., 2021; Mkhonto & Hanssen, 2017; Nwakasi et al., 2021; Karungi et al., 2022). Nwakasi et al. (2021) also found that participants believed dementia was associated with people becoming victims of their own “evil deeds” or karma (p1453). Musyimi et al. (2021) had similar findings with participants believing that dementia was a consequence of neglecting a husband or loved one. However, it should be noted that dementia is not unique in this respect, Mkhonto and Hanssen (2017) state that the origin of all misfortune is traditionally held to be social, while Nwakasi et al., (2021) suggest that in traditional African belief systems there are no ‘natural’ illnesses.

Another belief among participants was that “dementia was a white people’s disease”, not present in the African (Mkhonto & Hanssen, 2017, p. 172) or Iranian (Antelius & Kiwi, 2015) communities. Similarly, participants in the Musyimi et al. (2021) study believed dementia affected wealthy people who lived in the cities.

Nwakasi et al. (2021) and Karungi et al. (2022) found communities in Africa held conflicting views about dementia with some attributing the symptoms of dementia to normal ageing as well as sickness and/or disease. Musyimi et al. (2021) noted that the view of dementia as normal ageing was shared among health professionals. Participants in the Musyimi et al. (2021:2809) study also attributed dementia to ’madness’.

Healthcare assistants from Korea, working in the USA, held differing perceptions about dementia; some participants thought that dementia could be attributed to certain personality characteristics such as being “obsessive” or “sensitive” (Lee et al., 2018, p. 17). Antelius and Kiwi (2015) found that some Iranian healthcare workers believed dementia was triggered by leaving country of birth. Several studies found that healthcare workers attributed dementia to depression, isolation, and loneliness (Antelius & Kiwi, 2015; Lee et al., 2018; Musyimi et al., 2021).

Participants in the Antelius and Kiwi’s (2015) study indicated that they related dementia to pre-existing character flaws or personality traits such as a vivid imagination. Brooke et al. (2019) compared attitudes of student nurses across three continents. Students from all backgrounds initially understood dementia as part of normal ageing.

### Theme 2: ‘Stigma derived from cultural perceptions has consequences for people living with dementia’

This theme demonstrates how cultural perceptions shape responses to dementia. There were overlaps between belief systems which contributed to a complex picture of cultural understandings (Diagram 1) Participants across studies held the belief that dementia is part of normal ageing, which resulted in acceptance of deterioration and minimal intervention, partly because of the concurrent belief that nothing could be done (Brooke et al., 2019; Karungi et al., 2022; Mkhonto & Hanssen, 2017; Musyimi et al., 2021; Nwakasi et al., 2021). This reflected reality; in many under-resourced nations there were minimal care pathways, facilities, treatment or support (Karungi et al., 2022; Mkhonto & Hanssen, 2017; Musyimi et al., 2021). Healthcare workers’ priorities were not always dementia-related; instead priorities were safety and hygiene (Karungi et al., 2022) or HIV and poverty (Mkhonto & Hanssen, 2017). Rather than seeking help for the symptoms, a hospital trip might be associated with family ‘dumping’ the relative with dementia there (Musyimi et al., 2021).

Supernatural explanations often held sway in the absence of other accounts of unusual behaviour, or sometimes existed concurrently with a belief in ‘normal ageing’ (Brooke et al., 2019; Karungi et al., 2022; Musyimi et al., 2021; Nwakasi et al., 2021). Most commonly this was called ‘witchcraft’ or ‘sorcery’ and, which caregivers suggested could lead families to consult witchdoctors (Musyimi et al., 2021; Nwakasi et al., 2021). Both seeking and receiving help were perceived as shameful (Mkhonto & Hanssen, 2017; Nwakasi et al., 2022; Musyimi et al., 2021). In addition to isolation and neglect, supernatural explanations led to further negative consequences for older women with dementia. Abuse associated with a belief in witchcraft included stoning, being locked up, sexually assaulted, beaten, and killed (Brooke et al., 2019; Mkhonto & Hanssen, 2017; Musyimi et al., 2021; Nwakasi et al., 2021). Fear that a grandmother was a witch had consequences for family carers, as siblings who might have contributed to finances and caring withdrew, thus increasing isolation for the person with dementia and further stress for those who remained (Nwakasi et al., 2021).

Stigma, whether related to age, madness or witchcraft, had serious consequences for people living with dementia and for their families and this was addressed in many of the papers (Antelius & Kiwi., 2015, Lee et al., 2018, Brooke et al., 2019, Musyimi et al., 2021; Mkhonto & Hanssen; Nwakasi et al., 2022; Antelius & Kiwi., 2015; Brooke et al., 2019). According to the professional care-givers, stigma might deter families from seeking support (Lee et al., 2018; Brooke et al., 2019; Nwakasi et al., 2021). It could also lead to neglect and ostracization from family and the community (Brooke et al., 2019; Musyimi et al., 2021) or alternatively to intense efforts to protect the individual and their family by providing such high-quality care that outsiders would not guess the nature of the problem (or suspect witchcraft) (Nwakasi et al., 2021). The choice to use a care home was also stigmatised among non-western carers and nurses working in western countries (Antelius & Kiwi, 2015; Brooke et al., 2019; Egede-Nissen et al., 2017; Lee et al., 2018). This was particularly noticeable when ‘matching’ carers (i.e. from the same or similar ethnic group to the service user), who believed in ‘filial piety’ (a duty of care to one’s parents and grandparents), expressed their views about families’ decision not to provide family-centred care (Antelius & Kiwi, 2015; Lee et al., 2018).

Filial piety as a cultural factor driving dementia care was identified explicitly (Antelius & Kiwi, 2015, Musyimi et al.; Brooke et al., 2019; Lee et al., 2018) and implicitly (Egede-Nissen et al., 2017; Hirata & Harvath, 2016; Mazaheri et al., 2017). While this belief system led some professional carers to hold judgemental attitudes towards families (Antelius & Kiwi, 2015; Brooke et al., 2019; Lee et al., 2018), for others it motivated provision of the highest quality of care, the care that they would want for their own grandparents (Egede-Nissen et al., 2017; Lee et al., 2018; Mazaheri et al., 2017). Sometimes study participants tried to compensate for the lack of actual family care with behaviour that they associated with family, such as being more affectionate (Mazaheri et al., 2017) and shopping for and cooking culturally significant food (Lee et al., 2018; Mazaheri et al., 2017). Isolation appeared to be perceived as both a cause (Hirata & Harvath, 2016; Lee et al., 2018; Musyimi et al., 2021) and a consequence (Mkhonto & Hanssen, 2017) of dementia, which drew a similar response in some carers, that of spending more time with their service-users, especially if they perceived the actual family to be neglectful (Mazaheri et al., 2017). Many carers used their perception of whether their work was good enough for their own grandparents as a barometer of work well done and so job satisfaction (Mazaheri et al., 2017). The behaviour was sometimes linked with a belief in ‘karma’ in that what they did for others would in turn reward them in their own old age (Antelius & Kiwi, 2015; Lee et al., 2018; Mazaheri et al., 2017). Conversely, apparent lack of filial piety was associated with care in residential facilities (Brooke et al., 2019).

Specific cultural values around respect, hierarchy and human connections appeared to lead South Korean and Japanese participants to feel ‘bound’ to their service-users in a relationship which designated them as facilitators of their service-users’ well-being (Hirata & Harvath, 2016; Lee et al., 2018). These two groups expressed huge commitment to the well-being of the people with dementia that they looked after. Hirata and Harvath’s (2016) participants were particularly determined to problem-solve for their residents. Commitment could extend to ‘taking the blame’ when service-users became agitated or upset (Egede-Nissen et al., 2017) partly because behaviour could be linked to rudeness from staff (Hirata & Harvath, 2016), perhaps confirmed in the shared finding that these studies’ participants were less inclined to respect autonomy and more inclined to take responsibility for safety through ‘kind coercion’. Commitment was echoed in Bentwich et al.’s (2018) findings, in which Arab healthcare workers’ behaviours appeared to reflect strong ethical beliefs about patients’ rights and intrinsic value as humans. These carers described going to great lengths to promote autonomy, comfort and dignity of those they cared for, but framed this as an entitlement of humans, rather than related to their own characteristics.

The medical model understanding in combination with a biopsychosocial approach (in which person-centred care was expected) appeared to influence the behaviour of many but not all nurses and carers working in western countries (Antelius & Kiwi, 2015; Bentwich et al., 2018; Brooke et al., 2019; Egede-Nissen et al., 2017). Migrant nurses working in western countries are not homogenous (Antelius & Kiwi, 2015); individuals within groups (both migrant and non-migrant) may hold opposing beliefs. Cultural values and practices were not static and varied according to participants’ religious backgrounds (Bentwich et al., 2018), location (Brooke et al., 2019) age (Antelius & Kiwi, 2015), whether urban or rural, educated or uneducated (Brooke et al., 2019; Nwakasi et al., 2021) and generational identity (Antelius & Kiwi, 2015). Brooke et al. (2019) recognised that cognitive dissonance may arise for student migrant nurses as they adjust to working in services which they may not feel are appropriate for the care of older people, alongside colleagues who may hold different perceptions of the nature of dementia. For example, nurses brought up within societies that value ‘filial piety’ may be disturbed by institutionalised rather than family-orientated care.

## Discussion

The purpose of this review is to explore the variety of understandings of dementia worldwide, with a view to improving the experiences of people living with dementia and potentially for those who care for them too. The intent is not to evaluate alternatives and identify then export one version of the truth about effective dementia care, or to compare others against the approach that has guided our own practice, but instead to learn from different perspectives. As Fletcher (2021, 1820) warns, ‘By not taking seriously black voices that contest white knowledge-claims, we diminish our collective intellectual armoury in the face of dementia, when we should be keen to engage with all available avenues and foster knowledge plurality’. Person-centred care has long been accepted as the ‘gold standard’ in western countries, yet poor care persists, as does the exploitation of healthcare workers and family caregivers (Biggs et al., 2019; Fletcher, 2021). This paper is the first to aim for a neutral stance that avoids centring a western perspective.

Four previous reviews (Brooke et al., 2018; Brooke & Ojo., 2019; Spittel et al., 2019; Yaghmour, 2021), explored diverse healthcare professionals’ and care workers’ perceptions of dementia. Brooke et al. (2018) and Yaghmour (2021) did so specifically in relation to the person-centred model, while Spittel et al. (2019) emphasised the negative consequences of supernatural explanations. Spittell et al. (2019) and Brooke and Ojo (2019) explored cultural belief systems in Southern Africa, focusing on ‘witchcraft’ as an explanatory model. Supernatural beliefs increase stigmatisation, social exclusion and abuse, reduce access to healthcare services and make the caring role more difficult. In South Africa the apparent gap in services meant that there was no alternative explanatory model with an aligned support system with benefits for people with dementia and their families.

Brooke and Ojo (2019) concluded that nurses could support pluralistic care provision, in which spiritual (although not witchcraft-based) beliefs could be adopted in Sub-Saharan Africa (SSA). The current review confirmed the findings of previous reviews by Spittle (2019) and Brooke and Ojo (2019) with spiritual beliefs, like ‘witchcraft’, ‘evil spirits’ or ‘punishment from God’ found to be very strong in SSA countries. Spittle et al. (2019) argued that people who hold these traditional beliefs appear influenced in their understanding of dementia, regardless of whether they live in their first or second homelands.

While in the United States and Europe populations are becoming more secular, this is not the case globally, and religion is very important to people in Africa, Latin America, the Middle East and South Asia (Pew Research Centre, 2022). Beliefs can be deeply rooted, stemming from religion, cultural norms, and shared experiences (White et al., 2021) and include both spiritual beliefs and fate beliefs, which for most people are perceived as adaptive, resulting in positive psychological outcomes, such as reduced distress, and improved emotional clarity and life satisfaction (Boden, 2015).

The previous reviews were conducted in the UK (Brooke et al., 2018; Brooke & Ojo, 2019) Germany (Spittel et al., 2019) and Saudi Arabia (Yaghmour, 2021). Many healthcare workers are economic migrants who bring their cultural beliefs to new homelands, where they are expected to replace their original belief system with a biopsychosocial understanding and sign up to deliver care based on person-centred theory (Egede-Nissen et al., 2017). However, there has been little research that aims to learn from, instead of, or in addition to, teaching international healthcare staff.

The papers in the current review included studies that related in a similar way to migrant nurses in a process of acculturation (Antelius & Kiwi, 2015; Brooke et al., 2019; Egede-Nissen et al., 2017; Lee et al., 2018; Mazaheri et al., 2017) but also included studies where nurses and healthcare workers explored their perceptions of dementia in the cultures in which they were raised (Brooke et al., 2019; Hirata & Harvath, 2016; Karungi et al., 2022; Mkhonto & Hanssen, 2017; Musyimi et al., 2021; Nwakasi et al., 2021). Some of the latter did so with western models of dementia care in mind, and indeed, as in Brooke and Ojo’s (2019) review, many study participants held concurrent conflicting views on causation.

Not all the papers identified a specific theory of causation. In the absence of an explanatory model, the emotions and behaviours of people with dementia could be interpreted through empathetic, respectful observations (Mazaheri et al., 2017; Lee et al., 2018; Egede-Nissen et al., 2017; Bentwich et al., 2018), which could lead to greater promotion of independence (Bentwich et al., 2018). However, with an understanding based on a negatively valued ‘othering’ identity (old, female, a witch, or mad) culturally unacceptable or ‘deviant’ dementia-related behaviours were often associated with stigma, shame and rejection (Antelius & Kiwi, 2015; Brooke et al., 2019; Lee et al., 2018; Nwakasi et al., 2021). It has been argued that others’ reactions to people thus labelled may contribute to the person showing more of the associated problematic behaviours, which then further affects their experience of living with the condition (Fletcher, 2021).

‘Othering’ and exclusion are not limited to cultural associations with spirituality within collectivist societies (Hofstede, 2001). Libert and Higgs (2022) explain how medicalisation leads to exclusion in a more individualist society focused on successful ageing. This might imply that it is best not to have a ‘dementia label’, at least in the global north. While the diagnostic process has been encouraged due to potential benefits associated with access to treatment, services and information (World Health Organisation, 2018), there may be benefits in ‘not-knowing’, such as the continuation of usual family relationships. Airth and Oelke (2020) suggest stigma may be internalised and lead older people not to access services, thus minimising their exposure to the stigmatised identity.

In this review, ‘normal ageing’ was a shared belief as a cause of dementia across cultures, often held in combination with other beliefs. It was the least stigmatised suggested cause of dementia, but it was not explicitly connected with social inclusion either. Liu et al. (2008) noted that ‘normal ageing’ as an explanation can both alleviate and exacerbate stigma. When memory problems are perceived as a natural part of old age, then a person might be more likely to continue to live in their usual place, whether independently or with support. Alternatively, perceiving dementia as natural means no assessment, treatment, (if available) advice, care or support (Alzheimer’s Disease International, 2021) and may even exclude an individual from some services (Biggs et al., 2019).

The cultural value of filial piety is associated with collectivist cultures (Hofstede, 2001) and was reflected in attempts to relate to people with dementia as if they were family. This has been called ‘fictive kin’ (Taylor et al., 2021) and was associated with either feeling obliged to care in ways which prioritised the well-being of those cared-for over those doing the caring, or going the extra mile, so that people with dementia felt connected and loved. However, staff providing a ‘fictive kin’ level of attention noted emotional and financial costs to themselves; this is congruent with previous research identifying the potentially exploitative nature of this type of relationship (Dodson & Zincavage, 2007).

A medical model understanding might be expected to be less judgemental and perhaps more inclusive. However, it was not protective, perhaps because of co-existing ageism-related stigma in western countries (Dahlke et al., 2021) and supernatural explanations in Southern countries (Brooke & Ojo, 2019). Libert and Higgs (2022) suggest the medical model understanding of dementia is divisive, because it separates an individual’s history into two: pre and post diagnosis, and separates older people, those ageing successfully from those not. A medical model understanding of dementia would suggest causative factors including genetics and inflammatory conditions, in combination with behaviours such as smoking, excessive drinking of alcohol, poor diet, poor dental hygiene and not taking sufficient exercise (Mulugeta et al., 2022). This understanding neglects the more influential social determinants of health, (Jeste, 2022) which could include social isolation, but also poverty and lack of access to health-promoting environments and lifestyles (Biggs et al., 2019).

Isolation has been identified as a contributing factor to dementia throughout the papers in this review and in the wider literature (Yu et al., 2020) which could imply that social inclusion is protective. Social inclusion in the sense of lower risk of isolation might also be more expected in countries sharing collectivist cultural norms (Hofstede, 2001). A broader understanding of the term social inclusion can include factors such as having access to healthcare resources, money, information, good jobs and education, factors which contribute to a lower risk of developing many conditions associated with ill health in old age, including dementia (Airth & Oelke, 2020). None of the studies in this review identified social determinants of health as contributing factors in the development of dementia.

Dementia was sometimes considered to have been caused by neglect but the associated stigma generated further exclusion. A complicating factor is that some of the changes associated with the condition can result in lack of confidence, social withdrawal and a low threshold of frustration (Jenkins & Feldman, 2018) which could alienate friends and family long before dementia is identified, setting in progress an isolating dynamic in relationships with others. Similarly, Biggs et al. (2019) highlighted the two-dimensional cause of social disadvantage and exclusion that impacts on people living with dementia, mostly identified as originating from others’ stigmatising beliefs behaviour and actions, and less so due the behaviour of people with dementia themselves, who might be perceived as burdensome.

Threads of ‘othering’ and of ‘blame’ run through the papers. ‘Othering’ is implied when participants note that people with dementia are from different ethnicities or geographical locations to their own or are perceived to break cultural norms in their behaviours. This is sometimes combined with blame, so not looking after a husband properly, getting involved in witchcraft, not taking responsibility for one’s health and isolating oneself can all be perceived as leading to dementia. Both folk and medical explanatory models had related dynamics involving some degree of judgment around breaking approved cultural norms, stigma, rejection and further isolation, whether in a care home in the global north or otherwise isolated from family in the community in the global south.

Stigmatisation prevents people from accessing services, impacts negatively on perceptions of caregiving, and leads to the marginalisation and exclusion of people with dementia (Spittle et al., 2019). Yaghmour (2021) suggested that nurses who lacked positive perceptions of people with dementia failed to maintain autonomy and/or dignity in their care. Biggs et al. (2019) explored the relationship between dementia and social exclusion and linked exclusion with poor staffing and training, pointless social activities, restraint, institutionalisation and poor physical environments. Most reviews on this topic recommended raising awareness through education which may help prevent stigmatisation and promote social acceptance and inclusion (Brooke & Ojo, 2019; Spittel et al., 2019; Yaghmour, 2021).

Values which promote individualism, which Hofstede (2001) identifies as a cultural value associated with North America and Europe, (reflected in practice as person-centred care, respect for personhood, promoting independence) may not resonate globally (Tieu et al., 2022). Hofstede (2001) noted that autonomy is associated with individualistic cultural values; collectiveness-orientated cultures would emphasise the value of collaborative or family decision-making, where an individual’s needs are secondary to the needs of the larger group. While this may feel alienating to a person accustomed to an individualistic outlook, a collective culture might be expected to have social inclusion ‘built-in’. From that perspective, individualism may feel self-centred. This is reflected in our findings that some participants from collectivist cultures had judgemental attitudes towards families who were seen not to have included their older people. An individualised approach neglects how most people in the world exist interdependently in networks with others, regardless of geographical location (O’Connor et al., 2022; Tieu et al., 2022). While Nolan’s relationship-centred care model (2004) acknowledges the importance of the family carer and professional, this does not encompass the wider community in a way which would be congruent for collectivist societies. The benefits of societal support include greater awareness and reduced stigma, easier access to support, and empowerment of people with dementia and their family members (WHO 2021). The global dementia-friendly communities initiative illustrates that this approach could be adopted more widely, including within more individualist societies (Alzheimer’s Disease International, 2017).

## Strengths and limitations

Quality appraisal was not used to reject or include papers, however most papers included in the review were high quality. Integrative reviews are sometimes criticised as being less systematic than other strategies (Cho, 2022). The authors addressed this by following the protocol and reporting the process transparently.

The review aimed to explore the range of cultural understandings of dementia held by nurses globally. The review was limited as the authors could only read papers written in English; universal translations of academic papers are required so that knowledge is not filtered through the English (and Spanish) languages only. In future this could be addressed by use of Artificial Intelligence for automatic translation of academic papers.

Despite the broad search terms for this review, there was limited original research exploring cultural beliefs about dementia in the western world. This might indicate the extent to which these beliefs are taken for granted or perhaps assumed to be ‘normal’ and unworthy of explanation, or perhaps an assumption that ‘culture’ is something that other people do.

## Recommendations and conclusions

The purpose of this review was to identify the broadest possible range of understandings of dementia for mutual learning and perhaps acculturation, to benefit people living with dementia and those who care for them. The explanatory models can be considered in relation to the impact on people living with dementia. So, for example, while it is impossible to put aside our culturally derived doubts about witchcraft as a cause of dementia, the evidence indicates that this belief system is the most disadvantageous in outcome and so we should recommend, in common with other reviewers, that it be challenged and replaced. Previous reviews indicated it should be replaced by a medical model understanding. However, in considering what the best alternative would be, there is not one belief system identified through this review that results in consistently higher care quality among family or professional caregivers. The papers highlighted how the medical model, bio-psycho-social model, citizenship/human rights model, fictive kin approach and normal ageing belief system all have some advantages, but also significant drawbacks in relation to insufficient positive treatment and care outcomes, often in conjunction with exploitation of caregivers.

The person-centred approach (Kitwood, 1997) was not specifically identified in conjunction with the bio-psycho-social model, as might be expected. This approach (underpinned in theory by beliefs about the needs of people with dementia and concerning the nature of relationships) may resonate, both theoretically and morally, most positively in the individualist cultures of the global north. While person-centred care is achievable (Terkelsen et al., 2020), sustained implementation can be hampered by insufficient resources, inadequate training and support on the ground, and insufficient support and remuneration for professional care givers (Dunkle et al., 2022; Franco et al., 2022), this despite Kitwood’s acknowledgement of the importance of meeting care workers’ needs for personhood and respect. Recent commentary on his work notes how Kitwood’s thoughts on well-being at work are increasingly put into practice through interventions that benefit employees’ health, and how people living with dementia mostly do so in the context of their own communities, supported by family carers (Keady & Elvish, 2019; Kitwood & Brooker, 2019).

Nolan et al. (2004) suggest that family carers are key to service-users’ well-being and that relationships between and meeting needs of all in the ‘triangle of care’ (Hannan, 2014) are key to good outcomes. This ‘Relationship Centred Care’ model (Nolan et al., 2004) is more congruent with a middle ground between individualist and collectivist values, and although not explicitly reflected in the reviewed papers, it best correlates with the findings of this review and could be broadened to include a construct related to empowerment within communities. This reflects the most positive perspectives explored in the papers and seems culturally congruent for communities, practitioners, family carers and people with dementia in high- middle- and lower-income countries.

Anti-dementia stigmatisation appears to be a global phenomenon which is expressed according to local cultural contexts. It is also significant that dementia treatment and care are under-resourced worldwide, an issue that Airth and Oelke (2020) ascribe to ageism and neoliberal values, in that the well-being of old people is considered an unworthy and unwise investment. Both people with dementia and carers are potentially vulnerable (Egede-Nissen et al., 2017), so any interventions for learning need to be matched by fair, respectful working conditions for paid and family carers and living conditions for people with dementia. The relationship-centred care model (Nolan et al., 2004) acknowledges workers’ need for economic and emotional security.

While emphasising that non-discriminatory cultural values and just working practices are essential, we suggest that education should focus on an outcome: the quality of life and experiences of people living with dementia, and those of their family members and healthcare workers, within a framework of broader community support. This approach reflects Nolan et al.’s relationship centred care model (2004) and the findings of this review. Social inclusion, in whichever environment and through locally culturally congruent mechanisms, should be prioritised, based on the Universal Declaration of Human Rights (United Nations, 1948). Further research is required to examine the usefulness of incorporating a relationship-centred care approach internationally.
